# Analysis of NFATc1 amplification in T cells for pharmacodynamic monitoring of tacrolimus in kidney transplant recipients

**DOI:** 10.1371/journal.pone.0201113

**Published:** 2018-07-23

**Authors:** Nynke M. Kannegieter, Dennis A. Hesselink, Marjolein Dieterich, Gretchen N. de Graav, Rens Kraaijeveld, Carla C. Baan

**Affiliations:** Department of Internal Medicine, Section of Transplantation and Nephrology, Rotterdam Transplant Group, Erasmus MC, University Medical Center Rotterdam, Rotterdam, the Netherlands; University of Kentucky, UNITED STATES

## Abstract

**Background:**

Therapeutic drug monitoring (TDM) of tacrolimus, based on blood concentrations, shows an imperfect correlation with the occurrence of rejection. Here, we tested whether measuring NFATc1 amplification, a member of the calcineurin pathway, is suitable for TDM of tacrolimus.

**Materials and methods:**

NFATc1 amplification was monitored in T cells of kidney transplant recipients who received either tacrolimus- (n = 11) or belatacept-based (n = 10) therapy. Individual drug effects on NFATc1 amplification were studied *in vitro*, after spiking blood samples of healthy volunteers with either tacrolimus, belatacept or mycophenolate mofetil.

**Results:**

At day 30 after transplantation, in tacrolimus-treated patients, NFATc1 amplification was inhibited in CD4^+^ T cells expressing the co-stimulation receptor CD28 (mean inhibition 37%; p = 0.01) and in CD8^+^CD28^+^ T cells (29% inhibition; p = 0.02), while this was not observed in CD8^+^CD28^-^ T cells or belatacept-treated patients. Tacrolimus pre-dose concentrations of these patients correlated inversely with NFATc1 amplification in CD28^+^ T cells (r_s_ = -0.46; p < 0.01). *In vitro* experiments revealed that 50 ng/ml tacrolimus affected NFATc1 amplification by 58% (mean; p = 0.02).

**Conclusion:**

In conclusion, measuring NFATc1 amplification is a direct tool for monitoring biological effects of tacrolimus on T cells in whole blood samples of kidney transplant recipients. This technique has potential that requires further development before it can be applied in daily practice.

## Introduction

Therapeutic drug monitoring (TDM) is routinely used to optimize tacrolimus (TAC) dosing after organ transplantation.[1–3] Traditionally, the TAC dose is adjusted based on whole blood pre-dose concentrations (C_0_), that have an imperfect relationship with the occurrence of acute rejection and adverse events, such as nephrotoxicity and infection.[4–10] A promising strategy to overcome the limitations of traditional pharmacokinetic TDM may be to measure the biological effects of immunosuppressive drugs (pharmacodynamics).

The primary biological target of TAC in T cells is the calcineurin pathway, of which the nuclear factor of activated T cells (NFAT) is one of the most important signaling proteins.[11] The NFAT family consists of 5 members: NFATc1 (NFAT-2), NFATc2 (NFAT-1), NFATc3 (NFAT-4), NFATc4 (NFAT-3) and TonEBP (NFAT-5).[12] NFAT molecules are key players in the immune response after transplantation and are involved in T cell development, activation, differentiation, as well as in the production of cytokines like interleukin (IL)-2.[11, 13, 14]

**Fig 1:**
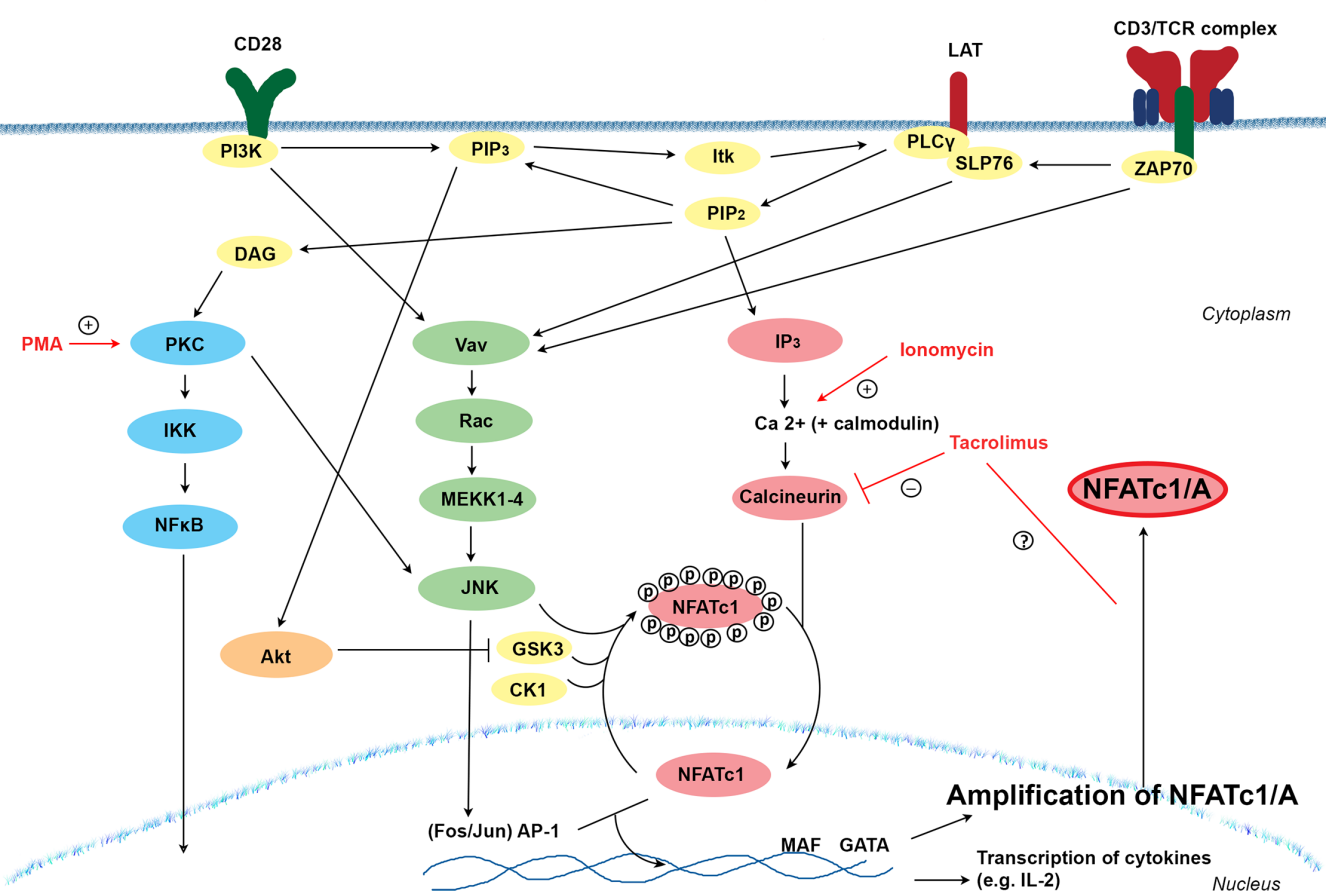
Schematic overview of the intracellular calcineurin pathway in T cells and amplification of the NFATc1/A isoform [11]. The calcineurin pathway is activated upon antigenic stimulation of the CD3/TCR complex in combination with co-stimulatory signals. This in turn activates the signal molecules phospholipase C-γ (PLC-γ) and inositol 1,4,5-trisphosphate (IP_3_) causing an influx of calcium and the opening of calcium channels in the membrane to maintain intracellular calcium levels. Upon interaction between calcium and the small calcium-binding protein, calmodulin, the phosphatase calcineurin is activated, which dephosphorylates NFAT. There are 13 phosphorylation sites present on NFAT that are known to be dephosphorylated upon activation. Dephosphorylation causes the translocation of NFAT to the nucleus where it will initiate gene transcription through the interaction with other transcription factors, such as AP-1. The signaling pathway is regulated by other signaling pathways, such as the MAPK pathway (JNK) and NFκB pathway. Once in the nucleus, NFAT will act as transcription factor and regulates the production of cytokines and the amplification of the isoform NFATc1/A in a positive autoregulatory feedback loop. The intracellular signaling pathways can also be activated by using PMA/ionomycin as a stimulus, while calcineurin inhibitors, such as TAC, are known to inhibit the calcineurin pathway.

Activation of the NFAT family member NFATc1 is initiated when both the T cell receptor (TCR) and co-stimulatory molecules, such as CD28, become activated (Fig 1). Upon activation, the phosphatase calcineurin is triggered, which then dephosphorylates NFATc1. In turn, dephosphorylated NFATc1 is translocated to the nucleus where it interacts with other transcription factors, such as AP-1, and induces gene transcription.

In contrast to other members of the NFAT family that are mainly known for their role in cytokine production, NFATc1 is also known for its strongly inducible isoform NFATc1/A. NFATc1/A is the only NFAT member that can be enhanced upon antigenic stimulation and maintained by positive autoregulation in T cells (Fig 1).[11, 15–19] The calcineurin inhibitor (CNI) cyclosporine A is known to inhibit both the dephosphorylation of NFATc1 and the upregulation of NFATc1/A, but the effect of tacrolimus on NFATc1/A amplification is still unknown.[20]

At present, clinically applicable pharmacodynamic assays to monitor the biological effect of TAC are in development, of which the NFAT-regulated IL-2 gene expression is the most promising. It was recognized that measuring NFAT-regulated genes might be a good method to assess the risk of opportunistic infections, malignancy and acute rejection after transplantation.[21–24] However, NFAT-regulated genes, such as IL-2, IFN-γ and GM-CSF are activated downstream in the calcineurin pathway. Subsequently, the activation of these genes can be influenced by other immunosuppressive drugs, such as glucocorticoids, and influenced by other signaling pathways, such as the JAK-STAT signaling pathway. [25, 26] Moreover, the measuring of NFAT-regulated gene expression is a non-validated tool to monitor the immunosuppressive effects in TAC-treated patients. A better way for monitoring the direct biological effects of tacrolimus might be the measurement of the immunosuppressive effect on NFATc1 amplification. Flow cytometry offers the opportunity to quantify the amplification of NFATc1/A at the single cell level with a short turnaround time when blood samples were spiked with cyclosporine A.[20] This technique enables the measurement of the immunosuppressive drug effects on the calcineurin pathway directly rather than measuring the end-products of this pathway, such as IL-2. Here, the applicability of the NFATc1 amplification assay is tested for the first time in whole-blood samples of TAC-treated kidney transplant recipients and we explored whether this method can be translated to daily clinical practice and can be an additional tool for monitoring the effects of TAC in different T cell subsets. Kidney transplant recipients, receiving a belatacept (BELA)-based maintenance therapy, served as a CNI-free control group, since BELA cannot directly inhibit the calcineurin/NFAT and other signaling pathways in T cells.[27, 28] In addition, NFATc1 amplification was also measured in different T cells subsets, *i*.*e*. CD4^+^ T cells, CD8^+^ T cells, CD28^+^ T cells, that need the expression of CD28 for co-stimulation and activation, and in the antigen experienced, potentially harmful, CD28^-^ T cells.[29, 30] Here, the difference between these cell subsets was measured to assess their susceptibility for immunosuppressive drug therapy.

## Materials and methods

### Kidney transplant recipients

Peripheral blood samples of 21 kidney transplant recipients were analyzed for the expression of NFATc1 in T cell subsets. The current study is a sub study of a prospective, randomized, clinical trial of which the results were published previously.[31] The study was approved by the Medical Ethical Committee of the Erasmus MC, University Medical Center (MEC number 2012–421, EudraCT # 2012-003269-16, registered October 17^th^ 2013) and samples were collected according to the biobank protocol that was also approved by the local ethics committee (MEC-2010-022). The work was performed in accordance with the Declaration of Helsinki. All patients gave written informed consent before the start of the study.

For this study, 21 of the total of 40 kidney transplant recipients that were randomized in the trial were included, since these patients were also analyzed for their NFATc1 expression before transplantation.[31] Of these, 11 patients received TAC-based and 10 patients received belatacept (BELA)-based immunosuppressive treatment. Samples from patients treated with BELA, that blocks the co-stimulatory CD80/86-CD28 pathway, were used as a control, because of the indirect effect of BELA on intracellular signaling pathways in T cells. Patients received a TAC starting dose (Prograf®, Astellas Pharma Inc., Tokyo, Japan) based on bodyweight (0.2 mg/kg/day) in two equally divided doses starting on the day of transplantation. Thereafter, the TAC dose was adjusted according to whole-blood pre-dose concentrations: 10–15 ng/mL (week 1–2), 8–12 ng/mL (week 3–4), and 5–10 ng/mL (from week 5 onwards). BELA (Nulojix®, Bristol-Myers Squibb, New York, USA) was given according to the less intensive regimen [32]: a dose of 10 mg/kg administered intravenously on day 0, 4, 15, 30, 60 and 90 after transplantation and then a reduced dose of 5 mg/kg as monthly intravenous infusions. All patients received an additional treatment consisting of mycophenolate mofetil (MMF; Cellcept®; Roche, Basel, Switzerland), prednisolone and basiliximab induction therapy (Simulect®, Novartis, Basel, Switzerland). MMF was administered at a starting dose of 1000 mg twice a day and then adjusted to pre-dose plasma concentrations between 1.5 and 3.0 mg/L. During the first three post-operative days, all patients received prednisolone intravenously in a dose of 100 mg/day. Thereafter, prednisolone was given orally in a dose of 20 mg/day and tapered to 5 mg/day by month 3 after transplantation. Basiliximab (20 mg) was given intravenously at day 0 and day 4 after transplantation.

### Blood samples and tacrolimus pre-dose concentrations

To measure the expression of NFATc1, blood samples were collected in heparin tubes (BD Biosciences, San Jose, CA) by venipuncture at days 0 (pre-transplantation), 4, 30, 90, 180 and 360 after transplantation and before anti-rejection therapy was started [in the case of an (suspected) acute rejection]. Samples were stored at room temperature on a tube-roller and on average prepared within 2 hours after venipuncture. Samples were not stored longer than 4 hours, to minimize the variability of NFATc1 amplification, due to the aging of blood. [33] TAC whole-blood C_0_ and mycophenolic acid (MPA) plasma C_0_ were measured in EDTA blood at the same time points by use of the antibody-conjugated magnetic immunoassay on a Dimension Xpand analyzer (Siemens HealthCare Diagnostics Inc., Newark, DE) according to the manufacturer’s instructions. The lower and upper limit of TAC detection were 1.5 and 30 ng/mL, respectively, and the coefficient of variation was 15.0%, 8.9% and 11.2% for the low, middle and high control samples, respectively. For MPA, the lower and upper limits of detection were 0.5 μg/ml and 15 μg/ml, respectively, and the coefficient of variation was 3.9% and 3.7%, for the low and high controls, respectively. Proficiency samples were obtained from the UK Quality Assessment Scheme (Analytical Services International Ltd, London, UK). Our laboratory successfully participates in the international proficiency testing program.

### Whole-blood intracellular staining for NFATc1

Heparinized blood samples were stimulated within 2 hours after blood collection with a final concentration of 0.5 μg/ml phorbol myristate acetate (PMA) and 10 μg/ml ionomycin for four hours at 37°C and in the presence of Golgiplug (BD Biosciences) to maximize the expression of NFATc1 and to induce NFATc1 amplification intracellularly.[20] Thereafter, 100 μl 20 mM EDTA was added to remove adherent cells from the activation tube and incubated for 15 minutes at room temperature. Samples were then stained with fluorescein isothiocyanate (FITC)-labeled mouse anti-human CD14 (clone UCHM1, Serotec, Oxford, UK), brilliant violet (BV) 510-labeled mouse anti-human CD3 (clone OKT3, Biolegend, San Diego, CA), peridinin chlorophyll (PERCP)-labeled mouse anti-human CD4 (clone SK3, BD Biosciences), allophycocyanin (APC)-Cy7-labeled mouse anti-human CD8 (clone SK1, Biolegend) and BV421-labeled mouse anti-human CD28 (clone CD28.2, BD Biosciences) for 30 minutes at room temperature. Subsequently, samples were lysed and fixed twice for 10 minutes with FACS lysing solution (BD Biosciences) and treated with permeabilization buffer II (BD Biosciences) for 10 minutes at room temperature. Phycoerythrin (PE)-labeled mouse anti-human NFATc1 (clone 7A6; Biolegend) was then added and incubated for 30 minutes on ice to determine the intracellular expression of NFATc1 in T cell subsets. Samples were analyzed on a FACS Canto II flow cytometer (BD Biosciences). Unstimulated samples were used to calculate NFATc1 amplification. An isotype control, mouse IgG1-PE, was included in a separate tube to see the background effect of antibodies binding on the NFAT molecule. Cytocalbeads (Thermo Scientific, Fremont, CA) were used to correct for interday-variability of the flow cytometer according to the manufacturer’s instructions. The conditions and concentrations used in this assay were established after optimization of the intracellular staining protocol in our lab. The intra-assay % CV values for NFATc1 amplification were 12.4%, 15.8% and 31.1% for the CD4^+^CD28^+^, CD8^+^CD28^+^ and CD8^+^CD28^-^ T cell populations, respectively.

### *In vitro* experiments

To measure the effect of the individual immunosuppressive drugs on NFATc1 amplification in T cell subsets, heparinized blood samples were drawn from healthy volunteers (n = 5). Samples were incubated for 16 hours overnight at 37°C with either vehicle (ethanol dissolved 1:8000 in distilled water), TAC (10 or 50 ng/ml), MPA (10 μg/ml; Sigma-Aldrich, Steinheim, Germany), prednisolone (100 ng/ml) or BELA (5 μg/ml), to be sure that the samples are well mixed with the immunosuppressive drug concentrations. Thereafter, samples were treated in the same way as the blood-samples of kidney transplant patients and stimulated for 4 hours with PMA/ionomycin at 37°C. After incubation, expression of NFATc1 was measured according to the protocol described in section 2.3.

### Statistical analysis

The median fluorescence intensity (MFI) of NFATc1 was measured and data-analysis was performed with Diva-version 6.0 software (BD Bioscience). MFI values were normalized using Cytocalbeads (Thermo Scientific). To calculate the total amplification of the inducible NFATc1/A isoform, samples were further analyzed by correcting the stimulated total expression of NFATc1 (both phosphorylated and dephosphorylated) for the MFI value in unstimulated samples (also both phosphorylated and dephosphorylated). Statistical analysis was performed with Graph Pad Prism 5.0 (Graph Pad Software Inc., La Jolla, CA) by using paired and unpaired t-tests (after finding a p-value > 0.05 with the Kolmogorov-Smirnov test for normality of the study population). Correlations between drug concentrations and the expression of NFATc1 were calculated as the Spearman correlation coefficient. A two-sided p-value < 0.05 was considered statistically significant.

## Results

### Patient demographics and clinical outcomes

S1 Table summarizes patient baseline characteristics, the incidence of rejection, and the medication of both TAC- and BELA-treated patients. In brief, the two study populations did not differ in their baseline characteristics. The incidence of rejection was lower in the TAC-treated group than in the BELA-treated group (1 out of 11 TAC-treated patients *versus* 7 out of 10 BELA-treated patients, respectively).[31, 34] In the current study, patients were censored from the moment of rejection onwards, since the expression of NFATc1 might be influenced by the anti-rejection therapy. Prednisolone doses were not significantly different between the two study groups, but the MPA pre-dose plasma concentrations were significantly lower in the TAC-treated group than in the BELA-treated group (p = 0.04). Further details regarding the clinical outcomes were published previously.[31, 34]

### NFATc1 amplification in T cell subsets

**Fig 2:**
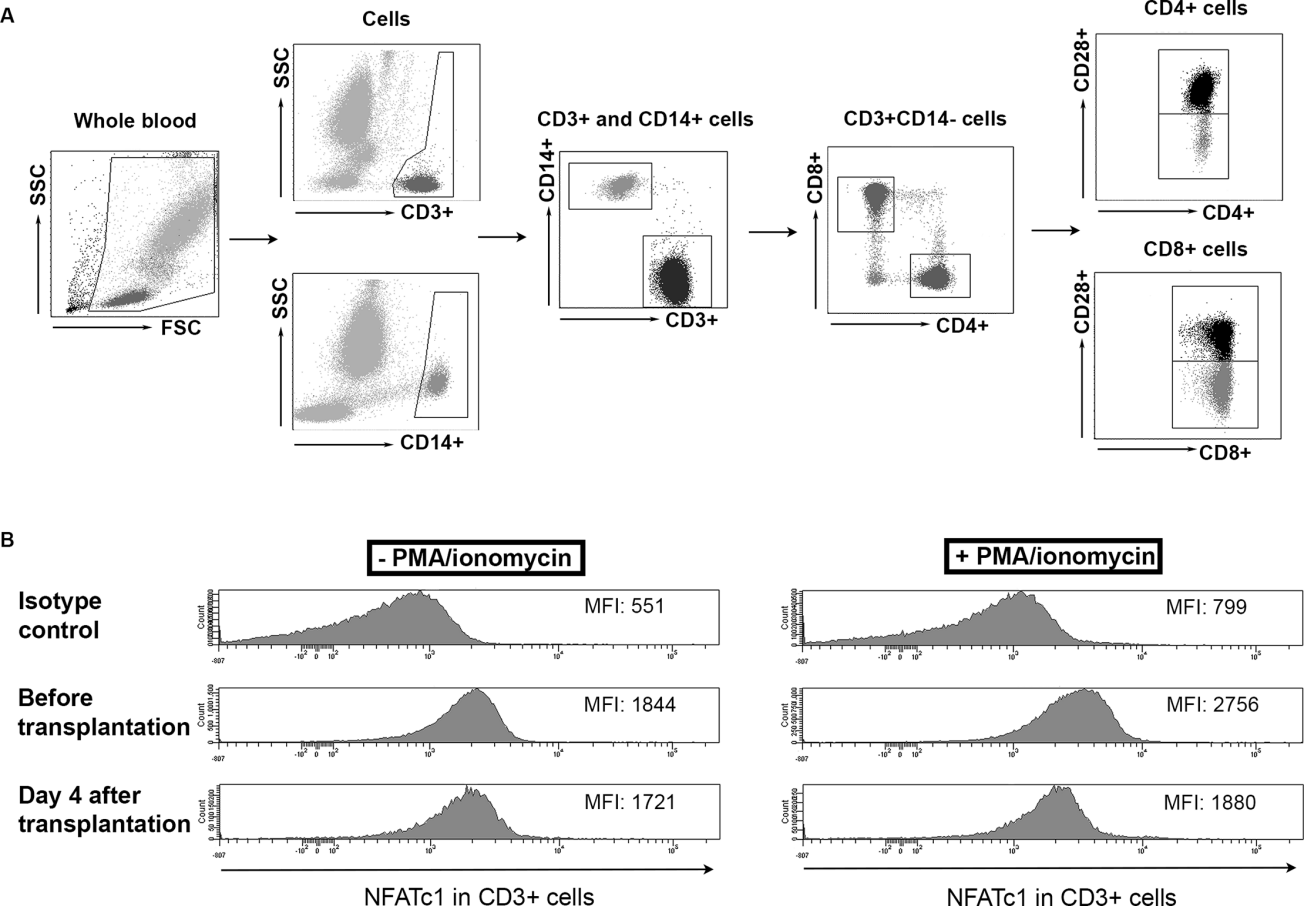
Gating strategy for the total NFATc1 expression in T cell subsets. (A) Gating of CD3^+^ T cell subsets. Cells were gated for their expression of CD14 and CD3, where after CD3^+^CD14^-^ T cells were gated for their expression of CD4 or CD8. Within these populations, the expression of CD28 was determined to identify the CD4^+^CD28^+^, CD8^+^CD28^+^ and CD8^+^CD28^-^ T cell subsets. **B)** Example of the total NFATc1 expression in CD3^+^CD14^-^ cells, either unstimulated or stimulated with PMA/ionomycin. FSC) Forward scatter; SSC) sideward scatter.

**Fig 3:**
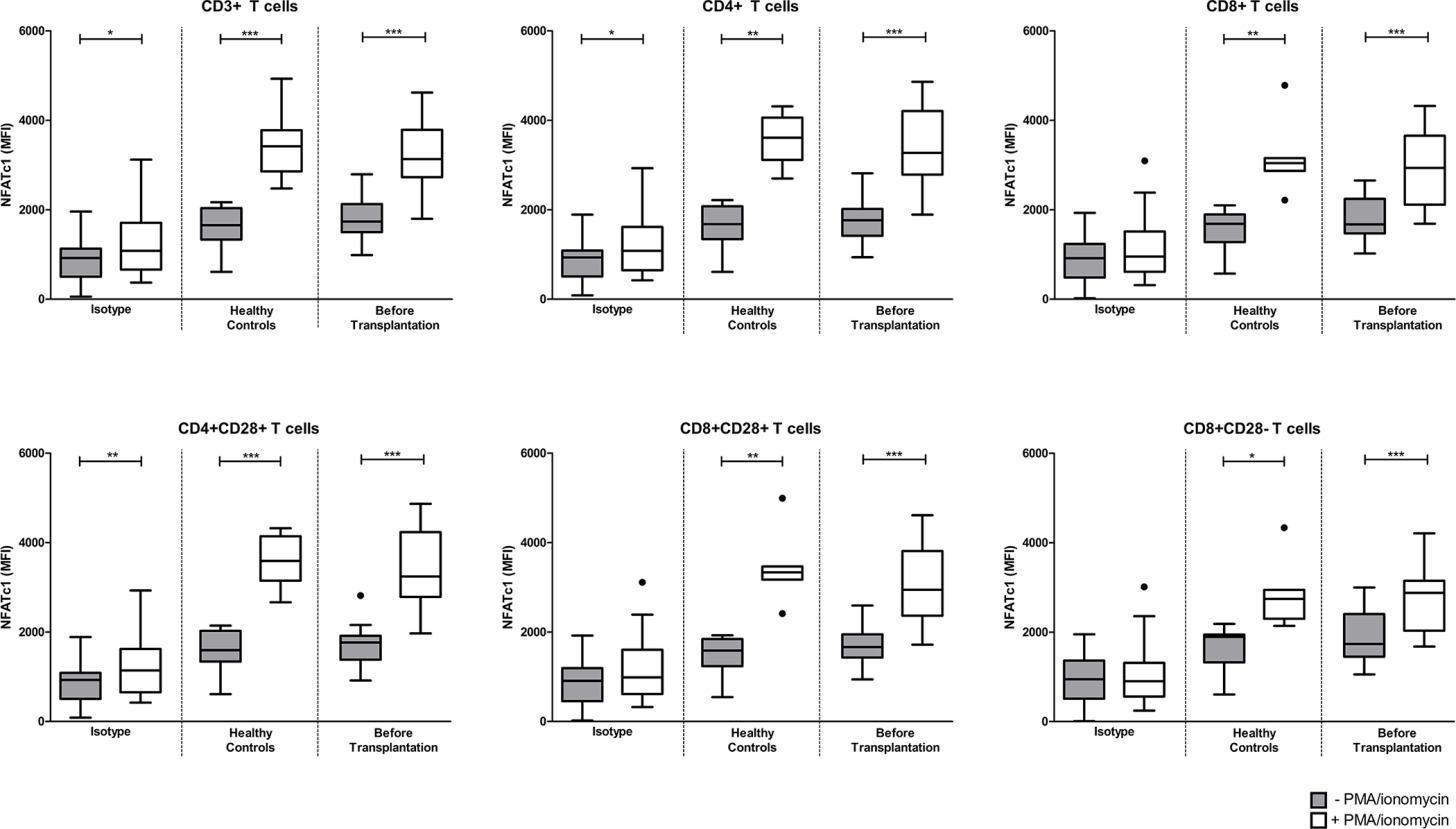
NFATc1 expression (MFI) in the total study population of TAC-treated patients. Unstimulated (*grey*) and PMA/ionomycin stimulated (*white*) blood samples were stained for the expression of NFATc1 in CD3^+^ (*upper left graph*), CD4^+^ (*upper middle graph*), CD8^+^ (*upper right graph*), CD4^+^CD28^+^ (*lower left graph*), CD8^+^CD28^+^ (*lower middle graph*) and CD8^+^CD28^-^ (*lower right graph*) T cells. Data are plotted as box and whiskers (Tukey style); n = 23 isotype controls, n = 10 healthy controls, n = 11 TAC patients before transplantation; *) p < 0.05, **) p < 0.01, ***) p < 0.001.

The effects of immunosuppressive drug therapy were determined in CD4^+^CD28^+^, CD8^+^CD28^+^ and CD8^+^CD28^-^ T cells. Upon stimulation, the expression of NFATc1 (expressed as MFI) increased compared to the unstimulated samples (Figs 2B and 3). After activation, no difference in the expression level of NFATc1 was found between healthy controls and patients before transplantation (Fig 3). Fig 2A shows the gating strategy for NFATc1 expression in CD3^+^ T cells.

**Fig 4:**
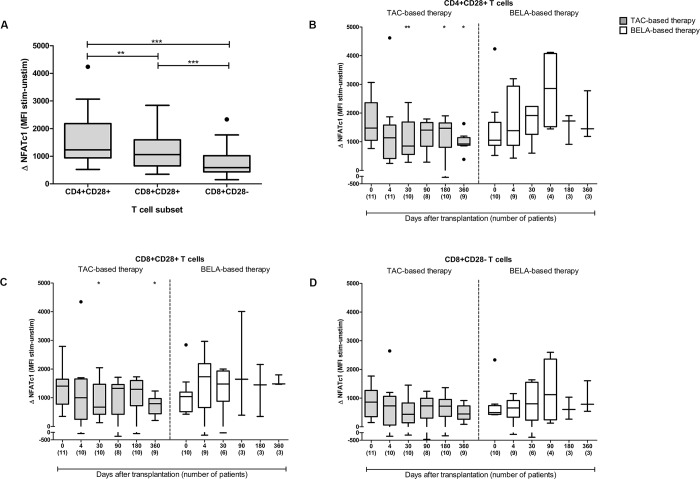
Immunosuppressive drug therapy effects on NFATc1 amplification in T cell subsets. (A) NFATc1 amplification in CD4^+^CD28^+^, CD8^+^CD28^+^ and CD8^+^CD28^-^ T cells before transplantation (n = 21 kidney transplant patients). (B, C, D) Inhibition of NFATc1 amplification (MFI) after a TAC-(*grey*) or BELA-(*white*) based therapy. Delta NFATc1 expression (amplification) was determined at different time points after transplantation and compared to the samples before transplantation in CD4^+^CD28^+^ (B), CD8^+^CD28^+^ (C) and CD8^+^CD28^-^ T cells (D). The number of patients that were measured at each time point is shown on the x-axis in parentheses. n = 11 TAC-treated patients and n = 10 BELA-treated patients at day 0. Data are plotted as box and whiskers (Tukey style); *) p < 0.05, **) p < 0.01 compared to day 0.

Before transplantation, the highest amplification of NFATc1 was found in CD4^+^CD28^+^ T cells, compared to CD8^+^CD28^+^ and CD8^+^CD28^-^ T cells (Fig 4A). After transplantation, in patients receiving TAC-based therapy, NFATc1 amplification was inhibited at day 30, 180 and day 360 in CD4^+^CD28^+^ T cells (mean inhibition of 37%, 24% and 28%; p = 0.01, p = 0.03 and p = 0.03, respectively; Fig 4B). In comparison to pre-transplantation, CD8^+^CD28^+^ T cells show a lower amplification of NFATc1 at day 30 and 360 after transplantation (mean inhibition of 29% and 15%; p = 0.02 and p = 0.03, respectively; Fig 4C**)**. In contrast, no effect of TAC-based therapy was observed in CD8^+^CD28^-^ T cells (Fig 4D). T cell subsets of BELA-treated patients were also not affected by the immunosuppressive drug therapy (Fig 4B–4D). However, as depicted in Fig 4B–4D, a wide range of NFATc1 expression was observed due to the small number of patients on belatacept treatment at day 180 and onwards.

### Correlation with TAC pre-dose concentrations

**Fig 5:**
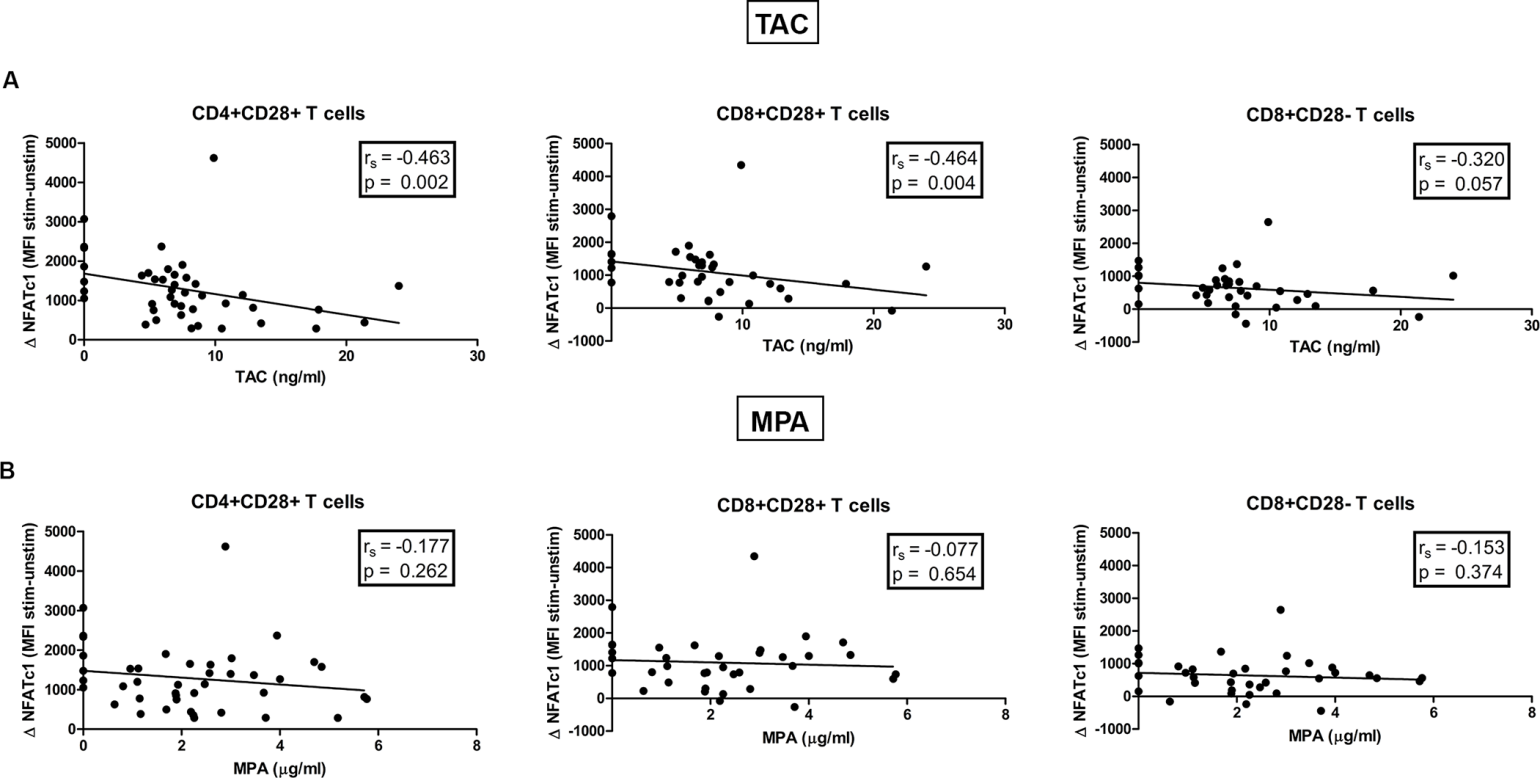
Spearman correlations of NFATc1 amplification with TAC or MPA pre-dose concentrations. (A) NFATc1 amplification inversely correlated with TAC pre-dose concentrations in time. The correlation was only seen in CD4^+^CD28^+^ (*left graph*) and CD8^+^CD28^+^ cells (*middle graph*) and not in CD8^+^CD28^-^ cells (*right graph*). (B) MPA pre-dose concentrations were not correlated to NFATc1 amplification levels in CD4^+^CD28^+^ (*left graph*) and CD8^+^CD28^+^ cells (*middle graph*) nor in CD8^+^CD28^-^ cells (*right graph*). TAC-treated patients were included for this analysis when blood samples were analyzed for NFATc1 amplification at all time points: before transplantation and day 4, 30, 90, 180 and 360 after transplantation. N = 7; r_s,_ Spearman correlation coefficient.

To determine whether TAC pre-dose concentrations correlated with the inhibition of NFATc1 amplification, correlations were calculated over time. For these calculations only samples from TAC-treated patients were included that were taken at each tested time point: day 0, 4, 30, 90, 180 and 360 (n = 7). Inverse correlations were found between TAC pre-dose concentrations and NFATc1 amplification in both CD4^+^CD28^+^ (r_s_ = -0.463; p < 0.01) and CD8^+^CD28^+^ (r_s_ = -0.464; p < 0.01) T cells, but not in CD8^+^CD28^-^ T cells (Fig 5A). No correlations were found between MPA pre-dose concentrations in TAC-treated patients and NFATc1 amplification for all three T cell subsets (Fig 5B).

### Immunosuppressive drug effect on NFATc1 amplification: *In vitro* study

**Fig 6:**
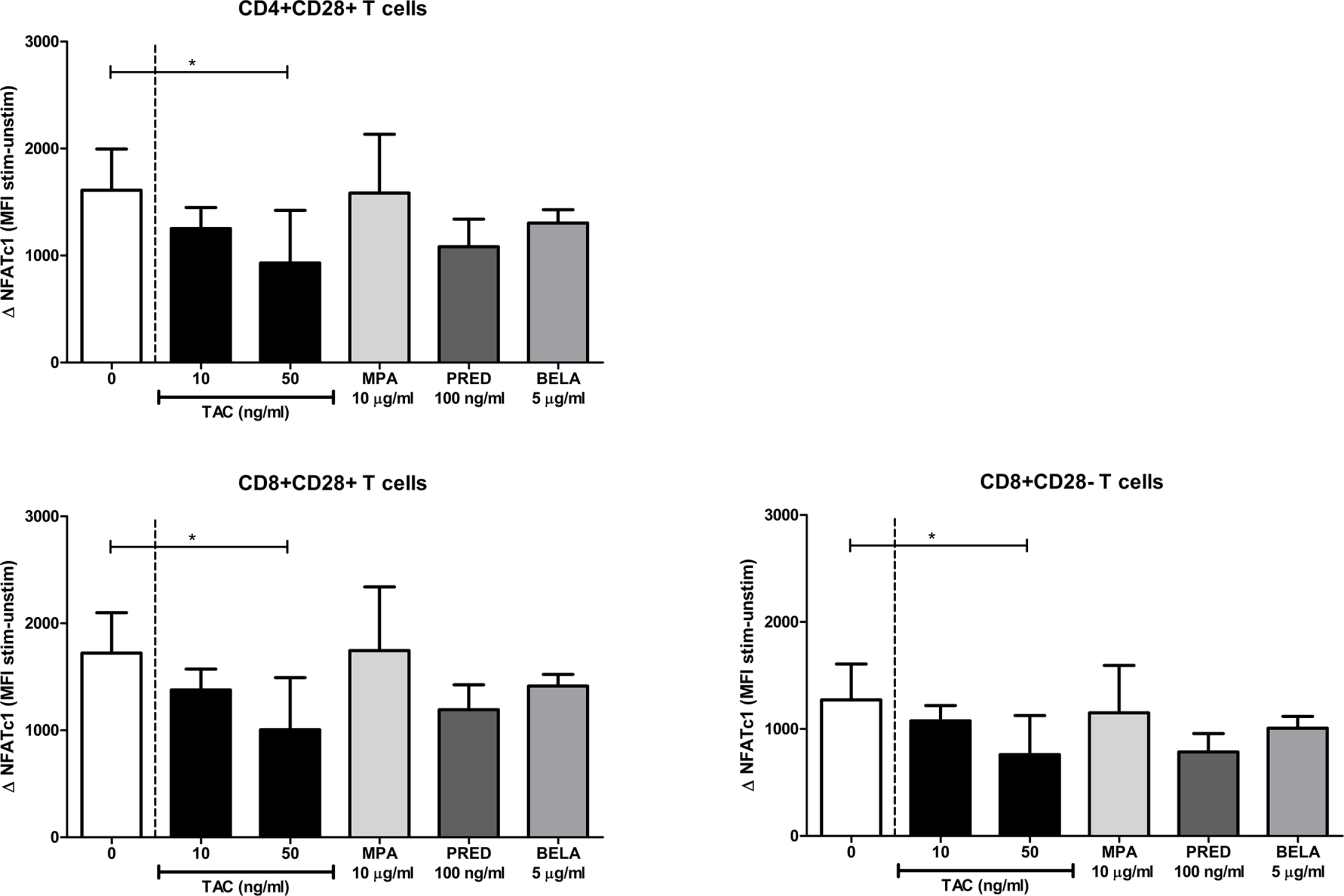
Individual drug effects on NFATc1 amplification in T cell subsets. TAC, at a high concentration of 50 ng/ml, significantly inhibited the expression of NFATc1 in both CD4^+^CD28^+^ (*upper graph*), CD8^+^CD28^+^ (*lower left graph*) and CD8^+^CD28^-^ cells (*lower right graph*) compared to the sample without drugs. Data are plotted as mean ± SEM; n = 5; *) p < 0.05.

Next, the individual immunosuppressive drug effects on NFATc1 expression were determined in blood samples of healthy controls to define whether TAC was the responsible drug for the observed effects on NFATc1 amplification in the studied patient samples (who used a combination of immunosuppressive drugs). At a concentration of 10 ng/ml TAC, a small, non-significant decrease in NFATc1 expression was noted in CD4^+^CD28^+^, CD8^+^CD28^+^ and CD8^+^CD28^-^ T cells (Fig 6). However, at a concentration of 50 ng/ml, TAC decreased NFATc1 expression (58%, 58% and 60% for the three T cell subsets; p = 0.02, p = 0.02 and p = 0.04, respectively), which is in contrast with the patient experiments which showed only an effect of TAC-based therapy on CD28^+^ T cells. MPA, prednisolone or the negative control BELA did not inhibit NFATc1 (Fig 6).

## Discussion

TDM after transplantation is necessary to avoid problems with the small therapeutic window of TAC. To date, measuring whole-blood TAC concentrations is the method of choice for TDM in most clinics.[2] However, these (pharmacokinetic) measurements are not ideal, due to their poor correlation with clinical outcomes, such as acute rejection.[6] An additional pharmacodynamic tool for TDM is needed that measures the biological effects of TAC on its direct targets, such as the calcineurin pathway member NFAT. Up until now, no clinically applicable pharmacodynamic assay is available to monitor the direct effect of TAC on NFAT expression after kidney transplantation, although several studies have attempted to develop an assay that measures NFAT-regulated genes.[35] For example, Maguire *et al*. studied the relation between TAC C_0_ and the translocation of NFAT to the nucleus.[36] Other studies have tried to correlate TAC C_0_ or spiked concentrations to the expression of NFAT-related genes.[37–39]

This study shows that the amplification of total NFATc1 expression, measured in whole blood samples by means of intracellular staining, is related to the pre-dose concentration of TAC after transplantation and is feasible. Upon stimulation of blood samples with PMA/ionomycin for 4 hours, only the amplification of the inducible NFAT isoform, NFATc1/A, was enhanced, which is consistent with previous studies.[20, 40] Under TAC-based therapy, both CD4^+^CD28^+^ and CD8^+^CD28^+^ T cells, which express the CD28 molecule (needed for co-stimulation and proliferation), showed an inhibition of NFATc1 amplification. This was not the case when patients were treated with BELA-based therapy, indicating that TAC is responsible for the reduced NFATc1 amplification. Furthermore, the correlation between TAC pre-dose concentrations and the magnitude of NFATc1 amplification in CD4^+^CD28^+^ and CD8^+^ CD28^+^ T cells indicates that TAC is an important factor for the decreased NFATc1 amplification in kidney transplant recipients. This correlation was not observed for MPA, which was also part of the combination therapy the patients received in this study. These results are in line with the previous findings of the *in vitro* study by Brandt *et al*., which showed that the other CNI, cyclosporine A, is also responsible for the inhibition of NFATc1 amplification.[20]

In contrast to CD28^+^ T cells, no effect of TAC-based therapy on NFATc1 amplification was noted in CD8^+^CD28^-^ T cells. These cells are known to be more antigen-experienced than their CD28-positive counterpart and are insensitive to immunosuppressive drug therapy that targets co-stimulatory molecules. However, up until now it has not been demonstrated that CD28^-^ T cells are also less responsive to TAC-based therapy.[29, 30] The overall amount of NFATc1 amplification was also lower in CD28^-^ T cells than in CD28^+^ T cells. An explanation could be that CD28 costimulatory signaling is needed for the inhibition of NFAT export from the nucleus back to the cytoplasm (deactivation).[41] In the presence of CD28, the signaling molecule glycogen synthase kinase 3 (GSK3) is inhibited, resulting in decreased phosphorylation of NFATc1 and, as a consequence, in the inhibition of nuclear export (Fig 1). Without CD28 expression, more NFATc1 molecules become phosphorylated and will be re-transferred to the cytoplasm, causing less amplification of NFATc1/A. However, in the current study, T cells were stimulated with PMA/ionomycin, which is a CD28-independent stimulation. This suggests that another and unknown mechanism may be responsible for the lower NFATc1 amplification in CD28^-^ T cells. TAC-based therapy had no effect on NFATc1 amplification in CD28^-^ T cells, confirming that these cells are indeed less sensitive to immunosuppressive drugs, probably due to the already low expression of NFATc1 in the nucleus. CD8^+^CD28^-^ T cells are also known for their immunoregulatory function, next to their more aggressive role in autoimmune diseases and malignancies, suggesting that the ineffective inhibition of these cells by immunosuppressive drug therapy could have a positive influence on graft survival after transplantation.[42, 43]

In line with the results found in patient samples, individual drug experiments also showed a decrease in NFATc1 amplification when a high concentration of TAC was used (50 ng/ml). However, in this setting, the high concentration of TAC also inhibited CD28^-^ T cells, although the total NFATc1 amplification was again lower than in the CD28^+^ T cells. MPA did not show any significant effect on NFATc1 amplification, whereas prednisolone seemed to have a small effect in some individual cases. This can be explained by the inhibitory effect of glucocorticoids on the transcription factor AP-1 via the glucocorticoid receptor.[44, 45] NFATc1 and AP-1 cooperate to induce gene transcription, for example the amplification of NFATc1. Without the function of AP-1, NFATc1 is less effective in the transcription of genes, which will cause the reduced NFATc1/A production.

The present study has limitations. First, the amplification of NFATc1 is still not the most direct way to measure the effects of TAC, although NFATc1/A is the only NFAT member of which the total expression can be induced after T cell receptor activation. Other factors can also regulate the induction of NFATc1, including the expression of AP-1.[46] The most ideal assay to monitor TAC exposure remains the measurement of NFAT dephosphorylation of all NFAT family members. However, since no such monoclonal antibody is available, the current assay can also provide sufficient information about TAC effects on NFAT functioning, since the induced NFATc1/A molecules contribute to the NFAT pathway as a positive feedback loop. Even more, the whole-blood assay presented here will give more sustained information about TAC exposure than assays performed with isolated T cells, because the tacrolimus concentrations are present during the whole stimulation procedure and are not washed away. In blood samples of transplant patients, we here showed that it is possible to measure the NFATc1 amplification in the presence of tacrolimus. This has clear advantages over isolated T cell procedures where tacrolimus and other immunosuppressive drugs are washed away during the T cell isolation process. Another advantage of the whole-blood assay is that the impact of immunosuppression can be determined in different T cell subsets, such as CD4^+^CD28^+^, CD8^+^CD28^+^ and CD8^+^CD28^-^ T cells. The assay is also drug specific, as is shown by the individual drug experiments. However, it remains unknown whether the other NFAT members that are present in T cells (*e*.*g*. NFATc2 and NFATc3) are also affected by TAC in the same way and to the same extent, since those NFAT members cannot be amplified.[11] Secondly, the small group size of this study might be a restriction for implementing the technique in daily clinical routine measurements. This is a single-center pilot study and it can only be recommended as a new method for biomarker monitoring when the individual responses to immunosuppressive drugs are validated.[21] For that a larger study population is needed to reveal the effect of baseline demographics on NFATc1 amplification. In addition, it should be studied whether gender or ethnicity have an influence on the NFATc1 amplification level. The association with these variables could not be analyzed in the present study because of the low number of females (2 of 11 patients) and non-Caucasians (1 of 11 patients). Furthermore, stimulation of T cells was achieved by means of PMA/ionomycin to ensure maximal NFATc1 amplification. An alloreactive, T cell receptor-based stimulus may also be investigated in future work as a more “physiologic” model to investigate the effects of immunosuppressive drugs in individual patients.

The NFATc1 amplification assay needs more validation before it can be tested and implemented clinically. Further standardization of the procedure will likely lower the large intra-assay variability observed in the present study. Future studies also need to focus on correlations between NFATc1 amplification and clinical outcomes, such as infection or subclinical rejection.[47] The present study included only one patient suffering from a rejection under TAC-based therapy, which makes it impossible to draw conclusions on the association between NFATc1 amplification and rejection risk. Other adverse events that occurred in this study were infections, urological problems, hematological problems and diabetes mellitus, but were not associated with a change in NFATc1 amplification (S1 Fig) The influences of adverse events on NFATc1 amplification will probably become clearer in a larger and prospective (multi-center) study. We feel that in order to optimize TDM of TAC, the pharmacodynamic measurement of NFATc1 as described here should be combined with classic pharmacokinetic TDM using the C_0_ or with alternative measurements. Measurement of the whole blood TAC area under the concentration *versus* time curve (AUC) or the intra-lymphocytic TAC concentration may give more information on the clinical relevance of the measured NFATc1 levels than the whole blood TAC C_0_, since 80% of TAC in the circulation is erythrocyte-bound where it is not pharmacologically active.[48]

In conclusion, measuring NFATc1 amplification is a direct method to determine the biological effects of TAC on divers T cell subsets in whole blood samples of kidney transplant recipients. This technique is feasible and has potential but requires further development before it can be applied in clinical practice.

## Supporting information

S1 Fig Adverse events in the tacrolimus-treated and belatacept-treated group. Samples from patients that suffered from an adverse event around a specific time point are shown in red for the 3 T cell subsets: CD4^+^CD28^+^ (left), CD8^+^CD28^+^ (middle) and CD8^+^CD28^+^ (right) T cells. Their corresponding NFATc1 expression is shown on the y-axis. Most adverse events occurred within the first week after transplantation. No association between adverse events and the measured NFATc1 amplification was observed.
(TIF)

S1 Table Summary of patient baseline characteristics, incidence of rejection and medication. (PDF)
