# Supplementary figures and images for: Analysis of NFATc1 amplification in T cells for pharmacodynamic monitoring of tacrolimus in kidney transplant recipients
Source: PLoS One. 2018 Jul 23;13(7):e0201113. doi: 10.1371/journal.pone.0201113 (PMC6056039; doi:10.1371/journal.pone.0201113)

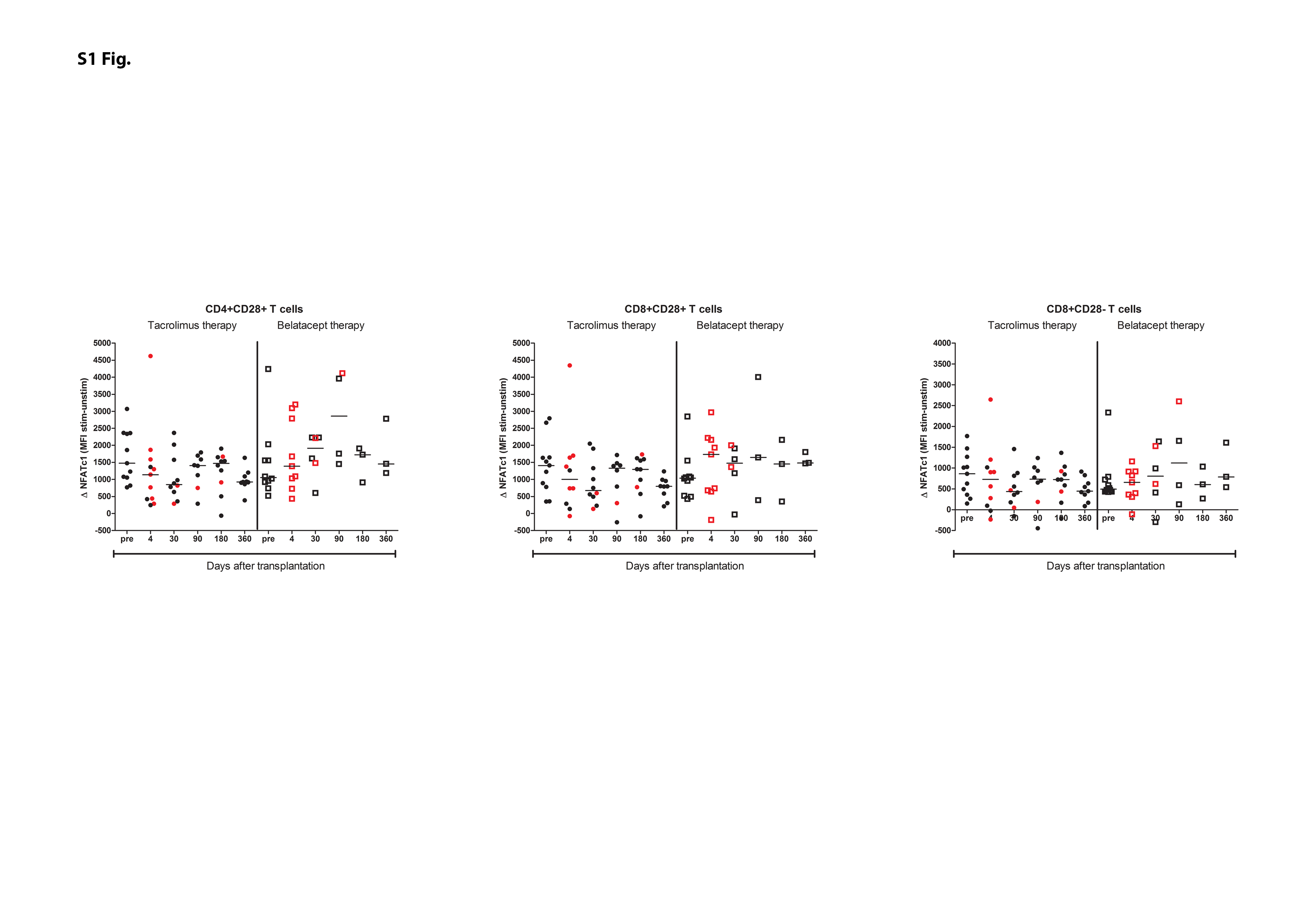

Supplement: S1 Fig — Samples from patients that suffered from an adverse event around a specific time point are shown in red for the 3 T cell subsets: CD4+CD28+ (left), CD8+CD28+ (middle) and CD8+CD28+ (right) T cells. Their corresponding NFATc1 expression is shown on the y-axis. Most adverse events occurred within the first week after transplantation. No association between adverse events and the measured NFATc1 amplification was observed. (TIF) [file pone.0201113.s001.tif]
