# Supplementary material for: Analysis of NFATc1 amplification in T cells for pharmacodynamic monitoring of tacrolimus in kidney transplant recipients
Source: PLoS One. 2018 Jul 23;13(7):e0201113. doi: 10.1371/journal.pone.0201113 (PMC6056039; doi:10.1371/journal.pone.0201113)
Supplement: S1 Table — (PDF) [file pone.0201113.s002.pdf]

S1 Table. Summary of patient baseline characteristics, incidence of rejection and medication

	Tacrolimus group (n = 11)	Belatacept group (n = 10)	p
**Age (years)**	55 (21-71)	46 (25-76)	*0.46*
**Male / female**	9 (82%) / 2 (18%)	6 (60%) / 4 (40%)	*0.30*
**Ethnicity**			*1.00*
• Caucasian	10 (91%)	9 (90%)	
• African	1 (9%)	1 (10%)	
**Body weight (kg)**	96.0 (63.3-103.0)	78.7 (56.6-111.4)	*0.10*
**Donor age (years)**	48 (22-80)	55.5 (39-70)	*0.46*
**BPAR (median time to rejection in days)**	1(152)	7(13)	
**TAC C_0 (ng/ml ±SD)**			
• Day 4	15.1 (± 4.8)	-	
• Day 30	9.9 (± 3.7)	-	
• Day 90	6.7 (± 1.7)	-	
• Day 180	6.7 (± 1.4)	-	
• Day 360	6.5 (± 2.0)	-	
**BELA dose (mg ±SD)**			
• Day 4	-	798 (± 180)	
• Day 30	-	740 (± 127)	
• Day 90	-	780 (± 180)	
• Day 180	-	358 (± 80)	
• Day 360	-	379 (± 69)	
**MPA C_0 (ng/ml ±SD)**			*0.04*
• Day 4	3.73 (± 1.52)	3.55 (± 1.10)	
• Day 30	2.89 (± 1.64)	3.67 (± 2.12)	
• Day 90	2.63 (± 0.94)	4.37 (± 1.76)	
• Day 180	2.16 (± 1.12)	2.78 (± 2.12)	
• Day 360	2.01 (±1.00)	1.99 (± 0.89)	
**PRED dose (mg ±SD)**			*0.16*
• Day 4	20.0 (± 0)	20.0 (± 0)	
• Day 30	12.7 (± 3.4)	11.6 (± 2.6)	
• Day 90	5.7 (± 1.6)	5.0 (± 0)	
• Day 180	5.0 (± 0)	5.0 (± 0)	
• Day 360	4.4 (± 1.1)	5.0 (± 0)	

Continuous variables are presented as medians (plus ranges) and categorical variables as numbers (plus percentages), unless otherwise specified

BPAR: The incidence of the first rejection episodes is given. The highest Banff score is depicted if sequential biopsies were performed.

BPAR, biopsy-proven acute rejection; C_0, predose concentration; MPA, mycophenolate mofetil; PRED, prednisolone; SD, standard deviation; TAC, tacrolimus
